# Buccal Ulcerations of Constitutional Origin

**Published:** 1882-03

**Authors:** 


### BUCCAL ULCERATIONS OF CONSTITUTIONAL ORIGIN.

---

Dr. Edward Wigglesworth, of Boston, read an interesting paper on these lesions at the last meeting of the American Dermatological Association. The following is an abstract of the paper:

These lesions are usually syphilitic in nature, though they are not always so. The fact that syphilitic ulcerations sometimes occur in persons above all suspicion of sexual immorality makes it doubly important for the general practitioner to recognize them by their appearance without asking too many questions. The only maladies likely to occasion doubt in regard to diagnosis are lupus and tuberculosis, and occasionally epithelioma. In cases of lupus the local treatment is identical with that adapted to ulcerations due to syphilis. When tuberculosis exists with ulcerations of the fauces or palate, or both, the pulmonary symptoms are almost always far advanced, and even when they cannot be detected, the patient is sure to die of tuberculosis, more or less general, and treatment is practically useless. The possibility, however, that the case may be one or either of these diseases should always make the practitioner careful in expressing an opinion and considerate in his language, for the peace of a whole family is often at stake.

The writer then went on to give notes of a series of illustrative cases, and followed these by some general remarks on the treatment, particularly of syphilitic ulcerations. Stimulants are better than caustics, and, indeed, much harm is done by the indiscriminate use of the latter. The parts must be kept well cleansed, and this alone when the ulceration is in the pharynx, gives great relief. The writer recommended the following solution for atomization :

℞ Tinct. iodini, pts. v ;
Glycerinæ, pts. x ;
Aquæ, pts. xxx.—M.

The most convenient instrument to use is that known as Fulgraffe's, which is made of hard rubber, with nozzles of various shapes—one to send a stream of spray up behind the soft palate, one for a horizontal stream, and one for use in the larynx. The bottle should be kept nearly full, so that the stream may not be kept up longer than is intended, owing to compressed air remaining in the bottle. Three or four applications of twenty seconds each will be sufficient to thoroughly cleanse and stimulate any ordinary ulceration.

After the use of the spray, iodoform should be applied to the ulcer with a camel's hair pencil, or blown on to it by an insufflator. A little powdered gum arabic mixed with the iodoform causes it to adhere better. Iodoform can also be conveniently deposited on the nasal mucous membrane by letting the patient inhale a saturated solution of the drug in ether.

Some cases require the use of the nasal douche with disinfectants. A convenient formula is one teaspoonful of chlorate of potassium with one-third as much carbonate of sodium and fifteen drops of a five-per cent. solution of permanganate of potassium, all in a quart of water at 25 degrees C., used with a fountain syringe. It is well to remember that a spray or a powder carried by a current of air may be used by blowing it into one nostril, while the patient breathes through the open mouth. The spray then passes through the vault of the pharynx and escapes by the other nostril. If desirable, by instructing the patient to breathe through the nose, a powder may be blown into the larynx through the nostril.

Constitutional treatment is of course necessary. The lesions occurring late in the disease—from the second to the twentieth year, and usually in cachectic subjects—iodine must play a more important part than mercury. Cod-liver oil, ferric iodide, potassic iodide, iodine spray, and iodoform—these are great remedies. Of course all hygienic measures must be united with these.—*Chic. Med. Journal and Examiner.*

